# Complete Genome Sequence of a *Pepper mild mottle virus* Isolate from Northeast China

**DOI:** 10.1128/genomeA.01500-17

**Published:** 2018-03-01

**Authors:** Man Yu, Tao Zhou, Yuanhua Wu, Mengnan An

**Affiliations:** a College of Plant Protection, Shenyang Agricultural University, Shenyang, China

## Abstract

The complete genome sequence of a *Pepper mild mottle virus* (PMMoV) isolate obtained from Northeast China was determined by reverse transcription-PCR (RT-PCR) and rapid amplification of cDNA ends (RACE). Phylogenetic analysis showed that the virus isolate is closely related to Japanese, Chinese, Spanish, and U.S. isolates.

## GENOME ANNOUNCEMENT

*Pepper mild mottle virus* (PMMoV) belongs to the genus Tobamovirus of the Virgaviridae family (1) and has a positive-sense single-stranded RNA with 6,356 nucleotides (nt). The genome of PMMoV encodes at least four proteins: a 126-kDa protein and a 183-kDa protein required for genome replication, and a 30-kDa movement protein (MP) and a 17.5-kDa coat protein (CP) expressed through subgenomic RNAs (2, 3). PMMoV is a major seed-transmitted viral pathogen on pepper (Capsicum spp.) (2–4), which was first reported in the United States (5). PMMoV causes mosaic symptoms and malformation on both the leaves and fruit of pepper and seriously reduces economical yields of crop (3, 4). In 2014, PMMoV caused great losses in Liaoning Province of Northeast China (6).

In this study, the complete genome sequence of the PMMoV isolate Huludao from Northeast China was obtained. The virus infectivity was confirmed through mechanical inoculation on pepper and Nicotiana benthamiana. Typical green mottle mosaic symptoms were observed on the upper leaves after inoculation. Total RNAs extracted from symptomatic leaves were used as the template for reverse transcription using the M-MuLV first-strand cDNA synthesis kit (Sangon, China). The sequences of the 5' and 3' ends of the virus were determined by 5' and 3' rapid amplification of cDNA ends (RACE) using a SMART RACE cDNA amplification kit (Clontech, USA). Reverse transcription-PCR (RT-PCR) was performed to amplify the complete genome sequence of the PMMoV isolate Huludao, with two pairs of primers, F1 (5'-GTAAATTTTTCACAATTTAACAACAACAAC-3') and R1 (5'-ACACCAAAGATTCGGAGCTC-3'), and F2 (5'-GCAGAGTCGCAGTGAGCTCC-3') and R2 (5'-TGGGCCGCTACCCGCGGTTC-3'). The PCR products (2,482 nt and 3,907 nt) were purified, cloned into the pMD19-T easy cloning vector (TaKaRa, Japan), and analyzed by DNA sequencing (Sangon, China).

A BLASTn search using the complete genome sequence of PMMoV isolate Huludao indicated that the isolate showed high identities (94.3% to ∼99.8%) with reported PMMoV sequences. The results indicated that the PMMoV isolate Huludao was closely related to other isolates, including those from Japan (GenBank accession no. AB000709, 99.8%), China (GenBank accession no. KP345899, 99.6%), Spain (GenBank accession no. M81413, 99.7%), and the United States (GenBank accession no. NC003630, 99.7%), whereas it was comparatively distantly related to isolates from Brazil (GenBank accession no. AJ308228, 94.5%) and South Korea (GenBank accession no. LC082100, 94.3%). Phylogenetic analysis was conducted using neighbor-joining methods with the program MEGA version 7 (7). The results indicated that the PMMoV isolate Huludao and Japanese, Chinese, U.S., and Spanish isolates clustered in one branch, which indicated that the isolates may have a common evolutionary ancestry. However, the Brazilian and South Korean isolates belonged to separate branches.

The import and export trades of seeds have become more frequent around the world, making it much easier for PMMoV to be transmitted by way of contaminated pepper seeds. PMMoV may cause fever, abdominal pain, and specific immune responses in humans (8), which suggests a significant association between the virus and food security. Therefore, more efforts are required for thorough quarantine inspection and the development of efficient virus-free pepper seed techniques. In conclusion, the study will assist further investigations on the infection and epidemiology of the virus in Northeast China.

### Accession number(s).

The complete genomic sequence of PMMoV isolate Huludao was deposited in GenBank under the accession no. MG515725.
